# Zika virus and host innate immunity: mechanisms of activation and evasion

**DOI:** 10.3389/fimmu.2026.1912541

**Published:** 2026-09-23

**Authors:** Danyu Qian, Yulei Guo, Aixin Li, Chunqiang Ma, Yanqi Zhao, Zhenpeng Li, Lixia Hui

**Affiliations:** 1 School of Laboratory Medicine, Shandong Second Medical University, Weifang, China; 2 Department of Clinical Laboratory, Affiliated Hospital of Shandong Second Medical University, Weifang, China

**Keywords:** antiviral signaling pathways, host restriction factors, innate immune evasion, virus-host interplay, zika virus

## Abstract

Zika virus (ZIKV) is an *Orthoflavivirus* that poses a significant public health threat due to its unique capacity to cause congenital malformations, neurological disorders, and reproductive complications. Upon infection, host cells sense viral RNA and danger signals through pattern recognition receptors, including Toll-like receptors, RIG-I-like receptors, inflammasomes and the cGAS-STING pathway, leading to the production of type I interferons and the subsequent expression of interferon-stimulated genes. Additional intrinsic restriction mechanisms, such as apoptosis, autophagy, RNA interference, stress granules, and nonsense-mediated mRNA decay, further limit viral replication. In response, ZIKV has evolved coordinated immune evasion strategies whereby multiple viral proteins target key signaling molecules—including RIG-I, MDA5, TBK1, IRF3, and STAT1/2—to subvert host antiviral defenses and promote viral persistence. This Review summarizes current knowledge of how the innate immune system recognizes ZIKV and how the virus counteracts these responses, while highlighting unresolved questions and potential therapeutic targets.

## 1 Introduction

First isolated in 1947 (1), Zika virus (ZIKV) remained largely neglected until its explosive emergence in the 21st century. The 2007 outbreak on Yap Island signaled its expansion beyond endemic regions (2), whereas the 2015–2016 epidemic in the Americas established a causal link between maternal infection and congenital microcephaly (3–5), transforming ZIKV from an obscure arbovirus into a pathogen of global concern. Since then, transmission has been documented in over 92 countries and territories, prompting the World Health Organization to designate ZIKV a priority pathogen (6). Despite its substantial clinical and societal burden, no licensed vaccine, virus-specific antiviral therapy, or routinely implemented prenatal diagnostic strategy is currently available (7).

ZIKV is an enveloped, positive-sense single-stranded RNA virus belonging to the genus *Orthoflavivirus* (8), with an approximately 10.7-kb genome encoding a single polyprotein. Following attachment of the viral E protein to host cell surface factors and receptor-mediated endocytosis, endosomal acidification triggers viral envelope fusion and release of the viral genome into the cytoplasm. The viral RNA is translated at the endoplasmic reticulum (ER) into a polyprotein, which is cleaved by host and viral proteases into structural proteins (C, prM/M, E) and nonstructural proteins (NS1, NS2A, NS2B, NS3, NS4A, NS4B and NS5). Within ER-derived replication complexes, the viral RNA-dependent RNA polymerase (RdRp) synthesizes negative-strand intermediates to generate progeny genomic RNA. Newly synthesized genomes are assembled with capsid proteins, bud into the ER, and mature through the Golgi apparatus before secretion. Importantly, the viral RNA intermediates and nonstructural proteins generated during replication are both sensed by and actively modulate innate immune pathways, establishing a direct mechanistic link between the ZIKV replication cycle and immune evasion (9–11).

ZIKV differs from other orthoflaviviruses in both its transmission routes and disease spectrum (12, 13). In addition to mosquito-borne spread by Aedes aegypti and Aedes albopictus, the virus can be transmitted sexually, vertically, and through exposure to contaminated body fluids (9, 14). This unusual transmission repertoire, together with the ability to cross the placental and blood-testis barriers, underlies congenital and reproductive complications, including congenital Zika syndrome (15) and impaired male fertility (16–18). ZIKV infection is also associated with post-infectious neurological conditions such as Guillain-Barré syndrome (19), which likely arise from aberrant immune responses rather than direct viral barrier breach. Collectively, these features suggest that ZIKV has evolved particularly effective strategies to evade antiviral immunity within specialized cellular environments.

The innate immune system constitutes the first line of defense against ZIKV infection. During this replication cycle, viral RNA and infection-associated danger signals are detected by Toll-like receptors (TLRs), Retinoic acid-inducible gene I (RIG-I)-like receptors (RLRs), inflammasomes, and the cyclic GMP-AMP synthase-stimulator of interferon genes (cGAS-STING) pathway, triggering the activation of interferon regulatory factors (IRFs) and nuclear factor-κB (NF-κB), and culminating in the production of type I interferons (IFN-I) and pro-inflammatory cytokines (20, 21). These responses induce hundreds of interferon-stimulated genes (ISGs) and are reinforced by intrinsic antiviral mechanisms, including nonsense-mediated mRNA decay (NMD), RNA interference (RNAi), stress granule (SG) assembly, ER stress and the unfolded protein response (UPR), apoptosis, and selective autophagy.

However, ZIKV has evolved a remarkably coordinated counteroffensive. Viral proteins antagonize nearly every step of innate immune signaling, from pathogen sensing to Janus kinase-signal transducer and activator of transcription (JAK-STAT)-mediated transcriptional responses, while simultaneously rewiring host intrinsic defense pathways to favor viral replication and persistence. Emerging evidence suggests that these interactions follow a convergent targeting strategy, in which multiple viral proteins repeatedly engage a limited set of signaling hubs, including RIG-I, melanoma differentiation-associated protein 5 (MDA5), TANK binding kinase 1 (TBK1), IRF3, and STAT1/2. This architecture confers both mechanistic redundancy and temporal flexibility, enabling ZIKV to fine-tune immune antagonism in a cell-type- and context-dependent manner.

In this Review, we examine the dynamic interplay between ZIKV and the host innate immune system through the lens of this convergent targeting model. We discuss how ZIKV is recognized by pattern recognition receptors (PRRs), how ISGs and intrinsic restriction pathways limit infection, and how viral proteins subvert these defenses. By integrating these findings into a unified conceptual framework, we highlight emerging principles of ZIKV pathogenesis and identify promising targets for antiviral intervention.

## 2 Host recognition and antiviral responses

### 2.1 PRRs for ZIKV

#### 2.1.1 TLR3 and TLR7/8

TLRs are a family of PRRs that recognize microbial components. Among the ten human TLRs, those localized intracellularly (TLR3, 7, 8, and 9) detect nucleic acids (21). TLR3 binds ligand and recruits the adaptor TIR-domain-containing adaptor inducing IFN-β (TRIF) (22), whereas TLR7 and TLR8 recruit myeloid differentiation primary response protein 88 (MyD88) (23). Both pathways activate IRFs and NF-κB, leading to IFN-I and pro-inflammatory cytokine production (23).

Following viral entry and endosomal trafficking, ZIKV RNA can be sensed by endosomal RNA sensors, including TLR3 and TLR7, with TLR8 also implicated in RNA recognition in specific immune cell contexts. TLR3 preferentially recognizes double-stranded RNA (dsRNA), including dsRNA intermediates generated during viral replication (24). Hamel et al. reported that ZIKV infection upregulates TLR3 expression in human skin fibroblasts (25), and Dang et al. made a similar observation in cerebral organoids and neurospheres (26). Plociennikowska et al. found that in ZIKV-infected central nervous system cells, TLR3 recognizes viral dsRNA and increases pro-inflammatory cytokine production. However, TLR3 activation suppresses the antiviral response triggered by RLRs, indicating complex cross-regulation among recognition receptors and enhancing our understanding of ZIKV pathogenesis (27).

In contrast, TLR7 recognizes single-stranded RNA (ssRNA) and stimulates the production of inflammatory cytokines (28). Bello et al. reported that the G-content-rich ZIKV genome is readily recognized by TLR7’s site 1, whereas its abundant UG motifs strongly activate TLR8, enabling stimulation of both TLR7- and TLR8-mediated immune responses (29). Santos et al. reported that a single-nucleotide polymorphism (rs179008) in TLR7 is associated with increased microcephaly severity in children with congenital Zika syndrome (30). From a therapeutic perspective, Vanwalscappel et al. showed that a TLR7/8 agonist significantly suppresses ZIKV replication (31). In summary, TLR3 and TLR7/8 contribute to the recognition of ZIKV RNA and shape the ensuing innate immune response. Their activation not only promotes antiviral and inflammatory signaling but may also influence disease severity through receptor crosstalk and host genetic variation. These insights clarify ZIKV pathogenesis and highlight targets for genetic susceptibility research and therapeutic development.

#### 2.1.2 RIG-I, MDA5 and LGP2

RLRs are a family of cytoplasmic PRRs that include RIG-I, MDA5, and laboratory of genetics and physiology 2 (LGP2). These receptors detect viral RNA and initiate innate immune responses (21). Upon detecting viral RNA, RIG-I and MDA5 undergo conformational changes and expose their caspase activation and recruitment domains (CARDs). These domains bind to mitochondrial antiviral signaling protein (MAVS) on mitochondria, which recruits TBK1 and IκB kinase ϵ (IKKϵ). These kinases then phosphorylate IRF3/IRF7, leading to their dimerization, nuclear translocation, and subsequent IFN-I production (32–35). Unlike RIG-I and MDA5, LGP2 lacks CARDs and does not directly activate MAVS (21). Instead, LGP2 regulates antiviral responses through RIG-I and MDA5. Early *in vitro* studies indicated that LGP2 negatively regulates RIG-I and MDA5 (36, 37), but subsequent studies using LGP2-deficient mice revealed that its ATPase domain promotes viral RNA recognition by RIG-I and MDA5 and enhances immune responses (38).

RIG-I and MDA5 recognize distinct types of viral RNA. RIG-I preferentially recognizes relatively short dsRNA, as well as 5′-triphosphate (5′-ppp) dsRNA or ssRNA, with the highest affinity for 5′-ppp dsRNA. In contrast, MDA5 preferentially recognizes longer viral dsRNA (39, 40). Accordingly, during ZIKV infection, RIG-I and MDA5 contribute to antiviral immunity through distinct recognition modes. Ma et al. reported that loss of RIG-I and MDA5 increases ZIKV replication and reduces IFN-β production (41). Schilling et al. observed that RIG-I serves as the primary RLR sensing ZIKV infection in A549 cells (42). Similarly, Jiyarom et al. reported that ZIKV induces an innate immune response via RIG-I and MDA5 signaling in Sertoli cells, which suppresses ZIKV infection (43), highlighting that their relative contributions are likely cell-type-dependent. Chazal et al. found that RIG-I activates the innate immune response by recognizing the 5′ end of the ZIKV genome (44). Furthermore, Hertzog et al. demonstrated that ZIKV infection leads to the accumulation of viral RNA that engages RIG-I via a 5′-phosphate-dependent mechanism, as well as RNA entities that activate MDA5 (45). These findings confirm that RLR signaling plays a critical role in inducing IFN-I and resisting ZIKV infection. LGP2, in contrast, acts through a different mechanism. Tan et al. demonstrated that ZIKV infection upregulates LGP2, which in turn inhibits ZIKV replication independently of IFN. LGP2 directly interacts with NS5, inhibiting its pre-elongation activity and exerting a unique antiviral effect (46). Thus, RIG-I and MDA5 primarily restrict ZIKV infection by sensing viral RNA and activating MAVS-dependent antiviral signaling, whereas LGP2 exerts an IFN-independent antiviral effect through direct inhibition of NS5. These findings suggest that RLR family members employ complementary mechanisms to limit ZIKV replication, integrating canonical innate immune signaling with direct interference in viral RNA synthesis.

#### 2.1.3 NLRP3 inflammasome

Nucleotide-binding oligomerization domain (NOD)-like receptors (NLRs) are intracellular sensors that detect microbial infection and cellular damage (47). Upon recognizing pathogen-associated molecular patterns, the NOD-like receptor family pyrin domain-containing 3 (NLRP3) assembles an inflammasome complex containing NLRP3, apoptosis-associated speck-like protein ASC, and the effector enzyme caspase-1. Activated caspase-1 cleaves pro-interleukin-1β/18 (pro-IL-1β/18) into their mature forms, leading to their secretion and initiating inflammation. Additionally, caspase-1 activation triggers pyroptosis, a process that plays a key role in host defense against pathogens (48–50).

In addition to direct viral recognition, host cells engage the NLRP3 inflammasome to sense infection-induced cellular stress and homeostatic perturbations. Infection-triggered danger signals, such as mitochondrial dysfunction, reactive oxygen species accumulation, ion imbalance, and lysosomal membrane permeabilization, converge to facilitate NLRP3 assembly and activation (51–53). Consequently, ZIKV-induced cellular stress likely synergizes with direct viral protein-mediated modulation to fuel NLRP3-driven inflammation. Wang et al. and He et al. demonstrated that ZIKV NS5 directly interacts with NLRP3, enhancing inflammasome assembly, activating caspase-1, and promoting IL-1β maturation and secretion, thereby contributing to inflammation and tissue injury (54, 55). B-cell lymphoma-2 (BCL-2) is known primarily as an anti-apoptotic protein but also negatively regulates NLRP3 activation, thereby inhibiting caspase-1 activation and IL-1β secretion (56, 57). Liu et al. found that ZIKV infection downregulates BCL-2 expression, relieving its inhibition of NLRP3 and leading to inflammasome activation and an exacerbated inflammatory response (58). In summary, NLRP3 activation during ZIKV infection involves both virus-induced cellular stress and viral protein-mediated regulation. ZIKV NS5 has been reported to promote NLRP3 inflammasome activation, potentially together with altered BCL-2-dependent regulation, leading to caspase-1 activation and pro-inflammatory cytokine maturation. These mechanisms may contribute to ZIKV-associated immunopathology and tissue injury.

#### 2.1.4 The cGAS-STING pathway

The cGAS-STING pathway plays a critical role in host innate immune defense against viruses. The cGAS detects cytoplasmic double-stranded DNA (dsDNA) and synthesizes cyclic GMP-AMP, which binds to ER-localized STING. STING then translocates to the Golgi apparatus and recruits TBK1. TBK1 phosphorylates IRF3, leading to its dimerization and nuclear translocation, followed by subsequent IFN-I gene transcription (59–63). Aguirre et al. showed that in dengue virus (DENV) infection, mitochondrial DNA (mtDNA) release triggers a cGAS-dependent IFN-I response (64). Similarly, Zheng et al. reported that ZIKV infection causes mitochondrial damage and mtDNA release, thereby enhancing cGAS-mtDNA binding and activating antiviral responses (65) (**Figure 1**). Collectively, although ZIKV is an RNA virus, infection-induced mitochondrial damage can promote the release of mtDNA into the cytosol and activate the cGAS-STING pathway. This mechanism links organelle dysfunction to TBK1-IRF3-dependent IFN-I responses, thereby broadening the scope of host immune surveillance against RNA virus infection.

**Figure 1:**
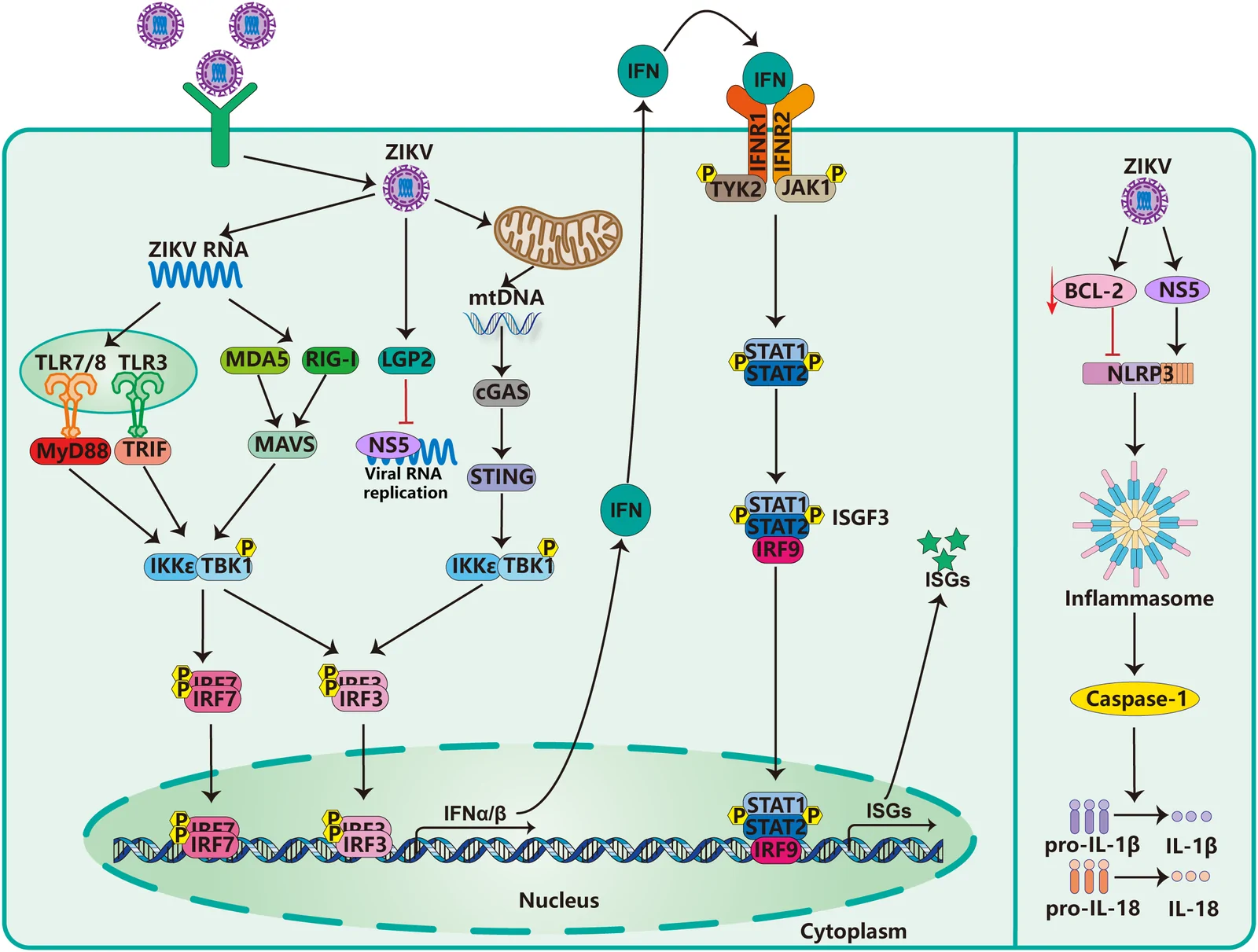
Recognition of ZIKV by PRRs induces IFN-I-mediated antiviral responses. TLR3 and TLR7/8 detect ZIKV RNA, respectively, activating IRF3/7 via TRIF and MyD88 pathways. RIG-I and MDA5 recognize viral RNA and, through MAVS, stimulate the TBK1/IKKϵ-IRF3/7 signaling cascade. LGP2 directly inhibits the transcription of ZIKV RNA mediated by ZIKV NS5. The cGAS-STING signaling pathway is activated by mtDNA released in response to ZIKV infection. ZIKV infection activates the NLRP3 inflammasome via NS5-NLRP3 binding and BCL-2 downregulation, thereby promoting the maturation and secretion of IL-1β and IL-18.

In summary, ZIKV activates multiple PRR pathways—including TLR3/7/8, RIG-I/MDA5, NLRP3, and cGAS-STING—to trigger innate immunity, leading to IFN-I and inflammatory responses. These receptors recognize distinct infection-associated signals in different cellular compartments: endosomal TLRs detect internalized viral RNA, cytosolic RLRs sense replicating viral RNA, NLRP3 responds to viral proteins and cellular stress, and cGAS detects mtDNA released following mitochondrial injury. However, complex cross-regulation exists among these pathways: for instance, TLR3 suppresses RIG-I signaling as noted above, whereas LGP2 directly inhibits viral replication independently of IFN. Understanding the balance between synergy and antagonism within these networks is crucial for uncovering ZIKV pathogenesis and developing targeted therapies.

### 2.2 ISGs with anti-ZIKV activity

ISGs are effector molecules downstream of the JAK-STAT signaling cascade that establish a multilayered defense against viral infection. During ZIKV infection, a variety of ISGs are markedly upregulated and contribute to antiviral defense through diverse mechanisms spanning multiple stages of the viral life cycle, from restricting early viral entry and translation to promoting viral protein degradation and modulating host signaling pathways (**Table 1**). It is important to note that while these pathways highlight major documented host defense strategies, they do not represent an exhaustive list, as additional ISG-mediated restriction mechanisms continue to be characterized.

**Table 1:** Summary of ISGs involved in host defense against ZIKV.

ISG	Target	Mechanism of action	Reference
IFITM3	ZIKV (early stage)	Inhibits viral entry (membrane fusion)	(66–68)
Viperin	eIF2α	Inhibits viral RNA translation	(69, 70)
	NS3	Degrades via the proteasome pathway	(73)
PARP12	NS1, NS3	Degrades via ubiquitin-proteasome system	(71)
TRIM22	NS1, NS3	Degrades via ubiquitin-proteasome system	(72)
C19orf66	NS3	Degrades via the lysosomal pathway	(74)
TRIM38	NS3	Degrades via the lysosomal pathway	(75)
OAS2	RIG-I	Activates RIG-I and increases IFN-β	(76)
	STAT1	Enhances STAT1 phosphorylation, activates JAK-STAT	
TAP1	TBK1-IRF3	Promotes TBK1-IRF3 phosphorylation, enhances IFN-I	(77)
ISG15	USP18	Prevents NS5-mediated STAT2 degradation, restores IFN-I signaling	(78)
IFI44	IFN-β, STAT1	Promotes IFN-β expression, activates STAT1 phosphorylation, positive feedback regulator of the JAK-STAT pathway	(79)
OASL-IT1	p38 MAPK, IRF3, NF-κB	Activates p38 MAPK, IRF3, NF-κB, induces IFN and ISGs positive feedback	(80)

At the earliest stage of infection, several ISGs restrict viral entry. Interferon-induced transmembrane proteins (IFITMs) inhibit viral entry by altering membrane properties to block fusion, thereby suppressing the replication of various orthoflaviviruses (66). Savidis et al. demonstrated that IFITM3 acts after virus binding but before viral RNA entry, restricting early ZIKV infection (67). Similarly, Monel et al. demonstrated that IFITM3 inhibits the early stages of ZIKV replication (68). Following viral entry, ISGs can interfere with viral protein synthesis. Viperin, an ISG of the S-adenosylmethionine (SAM) superfamily, is induced by IFN and viral infection and exhibits antiviral activity against various orthoflaviviruses (69). Hsu et al. showed that viperin’s SAMase activity produces 3′-deoxy-3′,4′-didehydro-CTP, which induces ribosomal collisions and activates the eukaryotic initiation factor 2α (eIF2α) kinase GCN2 integrated stress response, ultimately inhibiting ZIKV RNA translation (70).

ISGs also inhibit ZIKV replication by promoting the degradation of viral nonstructural proteins. Li et al. identified PARP12, a member of the poly (ADP-ribose) polymerase (PARP) family, which promotes K48-linked ubiquitination of the NS1 and NS3 proteins through its ADP-ribosylation and enzymatic activities, leading to their proteasomal degradation (71). Zu et al. reported that TRIM22 targets NS1 and NS3 for ubiquitin-proteasome degradation via its E3 ubiquitin ligase activity (72). In addition to inhibiting viral translation, Panayiotou et al. demonstrated that viperin mediates the proteasome-dependent degradation of the NS3 (73). Wu et al. found that C19orf66 binds NS3 and promotes its lysosomal degradation (74). He et al. showed that TRIM38 degrades NS3 in a lysosome-dependent manner through its E3 ligase activity (75). Collectively, these ISG-mediated degradation pathways converge to inhibit ZIKV replication.

Beyond directly targeting viral entry, translation, and protein stability, ISGs can reinforce antiviral immunity by modulating critical components of the IFN signaling pathway. Liao et al. showed that 2′,5′-oligoadenylate synthetase 2 (OAS2) exerts antiviral effects by promoting IFN-β activation through upregulating RIG-I, IRF7, tumor necrosis factor-α (TNF-α), and IL-8, as well as activating the JAK-STAT pathway by enhancing STAT1 phosphorylation, interferon-stimulated response element (ISRE) activity, and ISG expression (76). Zhao et al. found that transporter associated with antigen processing 1 (TAP1), which is upregulated by IFN-α, enhances IFN-I production by promoting the TBK1-IRF3 axis phosphorylation (77). Espada et al. reported that ISG15 restores IFN signaling by stabilizing ubiquitin-specific protease 18 (USP18), which competes with NS5 for STAT2 binding, thereby preventing NS5-mediated STAT2 degradation (78). A recent study showed that ZIKV-induced interferon-induced protein 44 (IFI44) forms a positive feedback loop by promoting IFN-β production, STAT1 phosphorylation, and ISRE activity to enhance downstream ISG expression (79). In addition, long non-coding RNAs (lncRNAs) also play an antiviral role. The lncRNA OASL-IT1, which is upregulated by IFN, activates p38 mitogen activated protein kinase (MAPK), IRF3, and NF-κB to induce IFN and ISG expression, forming a positive feedback loop that enhances innate immunity (80).

In summary, these findings illustrate that ISGs act at multiple distinct stages of the ZIKV life cycle, including initial viral entry, RNA translation, viral protein degradation, and the positive feedback amplification of host IFN signaling. This organizational structure explains the host’s resilient antiviral control and underscores the therapeutic value of targeting network hubs rather than single effectors. As existing evidence captures only a subset of this multifaceted defense, future investigations employing single-cell profiling and functional genomics are warranted to map cell-type-specific ISG interactions and advance ZIKV antiviral development.

### 2.3 Cellular intrinsic mechanisms that restrict ZIKV

Beyond canonical innate immune signaling, ZIKV infection elicits diverse intrinsic cellular defenses in response to replication-associated stress. In a coordinated temporal sequence, these defenses encompass early RNA quality-control pathways such as NMD, RNAi, SG dynamics, ER stress and the UPR, autophagy, and apoptosis, all of which collectively restrict viral propagation.

#### 2.3.1 RNAi-mediated antiviral restriction

RNAi represents a highly conserved gene regulatory mechanism in eukaryotic organisms, facilitating gene silencing through the specific recognition of target messenger RNA (mRNA) by small RNA molecules, which then induce mRNA degradation or inhibit translation. The RNAi pathway is initiated by the ribonuclease III family enzyme Dicer, which processes long dsRNA or precursor microRNAs (miRNAs) into small interfering RNAs (siRNAs) or miRNAs. These small RNAs associate with Argonaute (AGO) proteins to form the RNA-induced silencing complex (RISC), which targets and degrades complementary viral RNA (81, 82).

Previous investigations have shown that RNAi acts as an antiviral defense in mosquito cells. Upon recognition of viral dsRNA, Dicer processes these molecules into virus-derived siRNAs (vsiRNAs), which are incorporated into the RISC complex to facilitate sequence-specific degradation of viral RNA (83). Xu et al. reported that in human neural progenitor cells (hNPCs), ZIKV infection activates the RNAi machinery, generating abundant vsiRNAs with antiviral efficacy comparable to that in mosquito cells. In addition, the RNAi enhancer enoxacin exhibits potent anti-ZIKV activity and prevents ZIKV-induced microcephaly (84). Poirier et al. found that antiviral Dicer (aviD), a truncated form of Dicer that coordinates antiviral RNAi by cleaving viral dsRNA, protects tissue stem cells from ZIKV infection (85) (**Figure 2A**).

**Figure 2:**
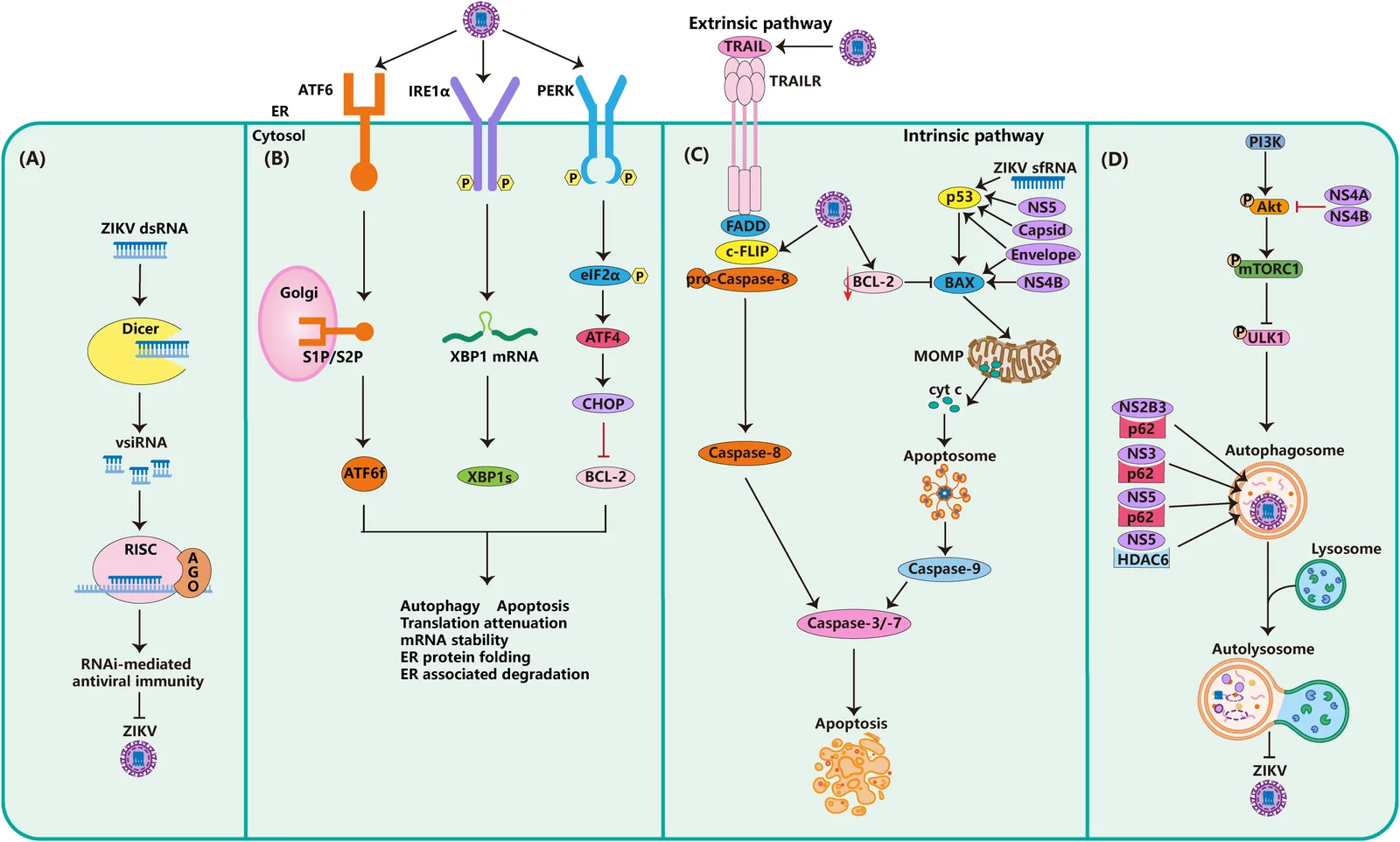
ZIKV infection triggers innate immune responses in host cells. **(A)** ZIKV infection activates RNAi, producing vsiRNAs processed and loaded into RISC to degrade viral RNA. **(B)** ZIKV-induced ER stress and UPR activation. ZIKV replication disrupts ER proteostasis and activates UPR signaling, including the IRE1α-XBP1, ATF6, and eIF2α pathways, with downstream effects such as CHOP-mediated apoptosis and impaired neurodevelopment. **(C)** ZIKV induces apoptosis via intrinsic and extrinsic pathways. In the intrinsic pathway, the C protein triggers p53-mediated apoptosis; the E protein upregulates p53 and BAX; NS4B binds and activates BAX; NS5 enhances p53 transcriptional activity; sfRNA induces p53-related pro-apoptotic gene expression; ZIKV downregulates BCL-2, leading to caspase-9 and caspase-3 activation. In the extrinsic pathway, ZIKV induces TRAIL-mediated signaling and alters c-FLIP function to promote caspase-8 and caspase-3 activation. **(D)** ZIKV stimulates autophagy by inhibiting the Akt-mTOR pathway via NS4A and NS4B, enhancing viral replication. Conversely, host promotes autophagic degradation of viral proteins: p62 mediates degradation of NS2B3, NS3 and NS5; HDAC6 targets NS5. This selective autophagy restricts ZIKV replication.

#### 2.3.2 ZIKV-triggered ER stress and UPR signaling

The UPR restores ER proteostasis through three canonical sensors: inositol-requiring enzyme 1 alpha (IRE1α), protein kinase RNA-like ER kinase (PERK), and activating transcription factor 6 (ATF6). Together, these branches coordinate translational attenuation, transcriptional reprogramming, and protein quality control. When ER stress is severe or prolonged, however, sustained UPR activation shifts from an adaptive response toward pro-apoptotic signaling. Mechanistically, PERK-mediated eIF2α phosphorylation suppresses *de novo* protein synthesis and facilitates the condensation of SGs. This coordinated axis functionally couples ER stress to cytoplasmic adaptation, establishing a crucial intrinsic barrier against viral exploitation of host translation (86–90).

Because ZIKV replication relies heavily on host ER compartments, extensive viral protein synthesis and extensive membrane reorganization disrupt ER proteostasis, provoking acute ER stress. Gladwyn-Ng et al. demonstrated that ZIKV-induced ER stress in the human fetal cortex, neural stem cells, mouse embryonic brains, and placenta serves as a primary driver of impaired neurodevelopment and microcephaly (91). Dissecting this response, Tan et al. demonstrated that infection robustly engages the IRE1α-X-box binding protein 1 (XBP1) and ATF6 axes in neural lineages (92), while Muthuraj et al. identified that ZIKV infection induces ER stress and engages UPR-associated pathways, including the IRE1α-XBP1 and eIF2α phosphorylation, while upregulating pro-apoptotic factor CHOP to promote apoptosis in placental trophoblast cells (93). A recent study has also revealed that ZIKV infection upregulates Glucose-Regulated Protein 78 (GRP78/BiP) expression and activates the UPR in A549 cells (94) (**Figure 2B**).

#### 2.3.3 Pro-apoptotic responses in infected cells

Apoptosis, a programmed form of cell death, plays a critical role in maintaining tissue homeostasis and host defense by eliminating senescent, damaged, or pathogen-infected cells (95). Upon detecting intracellular stress (e.g., DNA damage), the pro-apoptotic BCL-2 family member BAX is activated, causing cytochrome c (cyt c) release and mitochondrial outer membrane permeabilization. This leads to activation of caspase-9, which in turn activates caspases-3/7, triggering apoptosis via the intrinsic pathway (96, 97). The tumor suppressor gene p53 induces apoptosis through transcriptional activation of BAX expression (98). In the extrinsic pathway, upon binding of death ligands (e.g., tumor necrosis factor-related apoptosis inducing ligand (TRAIL)) to death receptors (e.g., TRAILR), fas-associated death domain protein (FADD) is recruited to form the death-inducing signaling complex, which activates caspase-8. Active caspase-8 then directly activates caspases-3/7, triggering apoptosis (96, 97). ZIKV activates host apoptotic pathways through multiple mechanisms, and this serves both as an antiviral response and as a basis for ZIKV pathogenesis.

Previous research has demonstrated that multiple orthoflaviviruses can induce apoptosis through intrinsic and extrinsic pathways (99). For instance, Japanese encephalitis virus (JEV) NS2B3 protease complex triggers intrinsic apoptosis via caspase-3 activation and cyt c release (100). West Nile virus (WNV) NS3 activates caspase-8 and caspase-3-mediated apoptosis (101). ZIKV employs similar strategies. For the intrinsic pathway, Teng et al. reported that ZIKV C protein interacts with the mouse double-minute-2 homolog, a regulator of p53-mediated apoptosis, thereby triggering neural death (102). Liu et al. found that in neuroendocrine cells, ZIKV E protein upregulates p53 and BAX, activating caspase-9 and caspase-3 and inducing apoptosis (103). Han et al. reported that in human neuroblastoma cells, ZIKV NS4B localizes to mitochondria, binds and activates BAX, leading to caspase-9 and caspase-3 activation and infected cell apoptosis (104). Li et al. showed that ZIKV NS5 binds the C-terminal domain of p53 via its methyltransferase (MTase) domain, thereby enhancing p53 stability and downstream transcriptional activity, which induces PARP cleavage and triggers apoptosis in hNPCs (105). Slonchak et al. found that ZIKV subgenomic flavivirus RNA (sfRNA) induces apoptosis in NPCs, impacting brain neural development, and that sfRNA production correlates with activation of pro-apoptotic genes including p53 (106). Additionally, Liu et al. demonstrated that ZIKV infection reduces anti-apoptotic protein BCL-2 expression, relieving its inhibition of apoptosis and increasing PARP and caspase-3-mediated renal cell apoptosis, establishing a link between ZIKV infection and kidney injury (56). Furthermore, Yuan et al. showed that the prM-S139N substitution enhances ZIKV infectivity and is associated with increased apoptosis and more severe microcephaly, although the extent to which apoptosis is a direct consequence of the mutation remains unclear (107).

For the extrinsic pathway, Lee et al. found that ZIKV induces TRAIL-mediated apoptosis in hNPCs, suppressing viral replication (108). Previous studies have reported that cellular FLICE-like inhibitory protein (c-FLIP) normally inhibits caspase-8 and caspase-10 activation, thereby suppressing apoptosis (109). In contrast, Zhang et al. found that during ZIKV infection in human trophoblast cells and murine-derived macrophages, c-FLIP upregulates caspase-8 and caspase-3, promoting apoptosis and viral replication as well as the development of congenital Zika syndrome. The molecular mechanism underlying this opposing effect may involve ZIKV infection altering the expression of c-FLIP subtypes, shifting the original inhibitory signal to a pro-apoptotic one, thus promoting virus-induced apoptosis (110) (**Figure 2C**).

#### 2.3.4 Dual role of autophagy in ZIKV infection

Autophagy is a cellular self-degradation and recycling process activated by oxidative stress or pathogen invasion. It sequesters aberrant proteins, damaged organelles, or viral particles within autophagosomes, which fuse with lysosomes to form autolysosomes for degradation (111, 112). Mechanistically, upon stress detection, protein kinase B (Akt) is dephosphorylated and inactivated, releasing its inhibitory effect on mammalian target of rapamycin (mTOR) and leading to mTOR inactivation. This prevents unc-51-like autophagy activating kinase 1 (ULK1) phosphorylation at its negative regulatory sites and thereby activating ULK1. Activated ULK1 then promotes the conversion of microtubule-associated proteins light chain 3 (LC3)-I to LC3-II via the ubiquitin-like conjugation system, and lipidated LC3-II mediates autophagosome elongation, target engulfment, and lysosomal degradation (113, 114), while sequestosome 1/p62 (SQSTM1/p62) serves as a selective autophagy receptor that bridges ubiquitinated cargo to LC3-II for selective clearance (115).

ZIKV infection enhances its replication and pathogenicity by activating autophagy. Liu et al. found that in human umbilical vein endothelial cells, ZIKV infection inhibited the Akt-mTOR pathway, suppressed ULK1 phosphorylation, and activated autophagy, promoting ZIKV RNA replication (116). Liang et al. showed that in fetal neural stem cells, NS4A and NS4B synergistically inhibit Akt phosphorylation, reduce mTOR phosphorylation, induce autophagy, and impair neurogenesis (117). ZIKV also exploits autophagy to remodel lipid metabolism. Lipid droplets serve as reservoirs for lipids essential for positive-strand RNA virus replication and participate in membrane biogenesis and energy metabolism (118). Stoyanova et al. demonstrated that early ZIKV infection elevates LC3-II levels, induces autophagy, and promotes lipid droplet formation, creating a favorable microenvironment for viral replication (119).

However, the host counters ZIKV by clearing viral proteins via selective autophagy. Huang et al. observed colocalization of autophagy markers (LC3, p62, LAMP1) with the ZIKV E protein in mouse hindbrain tissue, confirming autophagy in infected cells. ZIKV infection activates the IFN-α signaling, which is positively correlated with autophagy and may contribute to autophagic degradation of ZIKV, inhibiting viral replication (120). Wu et al. found that IFN-β facilitates the autophagic degradation of NS2B3 in a p62-dependent manner (121). Zhou et al. showed that p62 targets ZIKV NS3 and NS5 proteins for autophagic degradation (122). Silvia et al. reported that histone deacetylase 6 (HDAC6) mediates autophagic degradation of NS5, limiting its cytoplasmic accumulation and viral replication (123) (**Figure 2D**).

In summary, ZIKV activates multiple host intrinsic defenses: it induces RNAi-mediated viral RNA degradation, UPR, apoptosis, and promotes autophagy (while the host uses selective autophagy for viral clearance). Together, these mechanisms form a network that enables host cells to recognize and respond to infection. Regarding RNAi, although it inhibits ZIKV in both mosquito and human cells, its *in vivo* role in mammalian hosts with intact IFN systems remains unclear and requires further study. ZIKV replication heavily exploits the ER and triggers tripartite UPR pathways: while moderate PERK-eIF2α signaling acts as an intrinsic antiviral barrier by halting translation and promoting SG assembly, prolonged and decompensated ER stress drives CHOP upregulation and apoptosis, ultimately underlying microcephaly, neuropathology, and placental damage. In addition to the UPR, apoptosis is also particularly prominent in neural and placental cells following ZIKV infection, contributing to neurological disorders and placental pathology. Notably, c-FLIP—typically an apoptosis inhibitor—paradoxically promotes apoptosis during ZIKV infection, likely due to isoform switching, representing both a viral survival advantage and a therapeutic target. The role of autophagy in ZIKV infection is complex, and the seemingly contradictory effects reported in different studies likely reflect differences in viral strains, host cell types, infection stages, viral burden, experimental models, and autophagy-related processes. ZIKV may exploit autophagy-associated metabolic and membrane remodeling to facilitate replication, whereas selective autophagy pathways can contribute to antiviral defense by promoting recognition and degradation of viral components. Thus, the outcome of autophagy activation depends on the infection context, the specific autophagy pathways engaged, and the balance between proviral and antiviral effects. Uncovering the interplay among RNAi, UPR, apoptosis, and autophagy could lead to new combined therapeutic strategies for ZIKV.

## 3 Immune evasion strategies of ZIKV

### 3.1 Suppression of PRRs-mediated signaling

#### 3.1.1 Targeting the RLR signaling pathway

ZIKV extensively subverts the RLR pathway by targeting viral RNA recognition, adaptor transport, downstream kinases, and transcription factor activation. At the level of viral recognition and receptor transport, Sui et al. found that ZIKV prM protein binds MDA5, preventing MDA5-MAVS complex formation and suppressing IFN responses (124). Kim et al. showed that ZIKV NS1 interacts with RIG-I and MDA5, inhibiting RLR-mediated IFN signaling (125). Riedl et al. found that NS3 interacts with 14-3-3ϵ and 14-3-3η, blocking RIG-I and MDA5 transport from the cytoplasm to the mitochondrial membrane, thus suppressing IFN expression (126).

ZIKV also interferes with downstream components of the RLR pathway. Several studies have reported that NS2A and NS4A broadly inhibit multiple RIG-I/MDA5 pathway components, reducing NF-κB and IFN-β promoter activity (41, 127, 128). Hu et al. showed that NS4A directly binds MAVS and blocks its interaction with RIG-I via the CARD-transmembrane domain, suppressing IFN-I synthesis (129). Wu et al. reported that ZIKV NS1 and NS4B interact with TBK1 and inhibit IFN-β production by preventing TBK1 oligomerization (121). Xia et al. identified that NS2A, NS2B, and NS4B inhibit TBK1 phosphorylation, NS4A blocks IRF3 phosphorylation, and NS5 may bind IRF3 or modulate downstream signaling to impair IFN-β expression (130). Zhao et al. further found that NS5 accumulates in the nucleus via a nuclear localization signal and inhibits IFN-I production by blocking IRF3 activation (131). While ZIKV NS5 has been well documented to suppress RLR-mediated induction of IFN-I (42, 45), recent mechanistic studies demonstrate that this inhibition occurs through actions at distinct steps of the signaling cascade. Lundberg et al. demonstrated that ZIKV NS5 suppresses the expression and phosphorylation of IKKϵ, thereby dampening RIG-I pathway activity, blocking IRF3 phosphorylation, and ultimately reducing IFN promoter activation (132). Li et al. showed that NS5 directly binds the CARD domain of RIG-I, inhibiting its K63-linked ubiquitination and thereby blocking IRF3 phosphorylation and nuclear translocation, which suppresses IFN-β production (133).

In addition to directly targeting signaling proteins, ZIKV exploits host regulatory pathways to attenuate RLR signaling. Chen et al. found that ZIKV infection increases the 7-dehydrocholesterol reductase (DHCR7) expression in human astrocytes. ZIKV NS4B directly interacts with DHCR7, enhances DHCR7 expression, and impedes TBK1-mediated IRF3 activation, thus inhibiting innate immune signaling (134). ZIKV can also hijack selective autophagy to eliminate key antiviral signaling molecules. Lee et al. showed that ZIKV NS4A induces mitophagy by upregulating the mitophagy receptors BNIP3, NIX, and PINK1, thereby suppressing MAVS expression and IFN-I signaling (135). Zhang et al. demonstrated that ZIKV NS1 binds tumor necrosis factor receptor-associated factor 6 (TRAF6) and promotes its p62-dependent autophagic degradation, suppressing inflammatory factors and IFN-β (136). Yu et al. reported that ZIKV E, NS2A, NS3, and NS4B may cooperatively induce autophagic degradation of AXL and TIM-1, which reduces AXL-mediated IFN-β and ISG15 responses and prolongs viral replication (137).

#### 3.1.2 Targeting the cGAS–STING signaling pathway

In addition to RNA-sensing pathways, ZIKV potently antagonizes cytosolic DNA sensing mediated by the cGAS-STING axis through multiple distinct mechanisms. At the cGAS level, Zheng et al. found that NS1 recruits the deubiquitinating enzyme USP8 to stabilize caspase-1, which cleaves cGAS and disrupts the antiviral immune response (65). At the STING level, ZIKV employs a multi-layered inhibition strategy involving direct cleavage and proteasomal degradation. Ding et al. found that ZIKV NS2B3 cleaves human STING, disrupting cGAS-STING signaling and inhibiting IFN-I production. In contrast, murine STING is resistant to NS2B3-mediated cleavage due to key amino acid differences, highlighting the role of viral protease species specificity in viral host range (138). DENV, JEV, and WNV employ similar mechanisms (138, 139). Li et al. showed that NS5 interacts with STING and promotes its degradation via polyubiquitination, suppressing IFN-β production (140).

#### 3.1.3 Modulation of the NLRP3 inflammasome pathway

ZIKV also modulates the NLRP3 inflammasome, although the reported effects appear to vary according to viral strain, cell type, and experimental context. Li et al. reported that ZIKV SZ01 strain inhibits NLRP3 activation and cell death, a mechanism that may facilitate persistent infection and contribute to viral pathogenicity (141). Gim et al. found that in ZIKV MR766 strain-infected bone marrow-derived macrophages (BMDMs) and mixed brain glial cells, NS3 induces NLRP3 degradation, reducing caspase-1 activation and IL-1β secretion to facilitate immune evasion (142). However, as mentioned above, conflicting observations also exist. Wang et al. showed that infection with the ZIKV isolate z16006 promotes NLRP3 inflammasome assembly and IL-1β secretion in human peripheral blood mononuclear cells, macrophages, mice, and mice bone marrow dendritic cells (54). Similarly, He et al. confirmed this effect with the Asian lineage ZIKV SZ01 strain in BMDMs (55). This discrepancy may stem from differences in cell type or viral strain (**Figure 3**).

**Figure 3:**
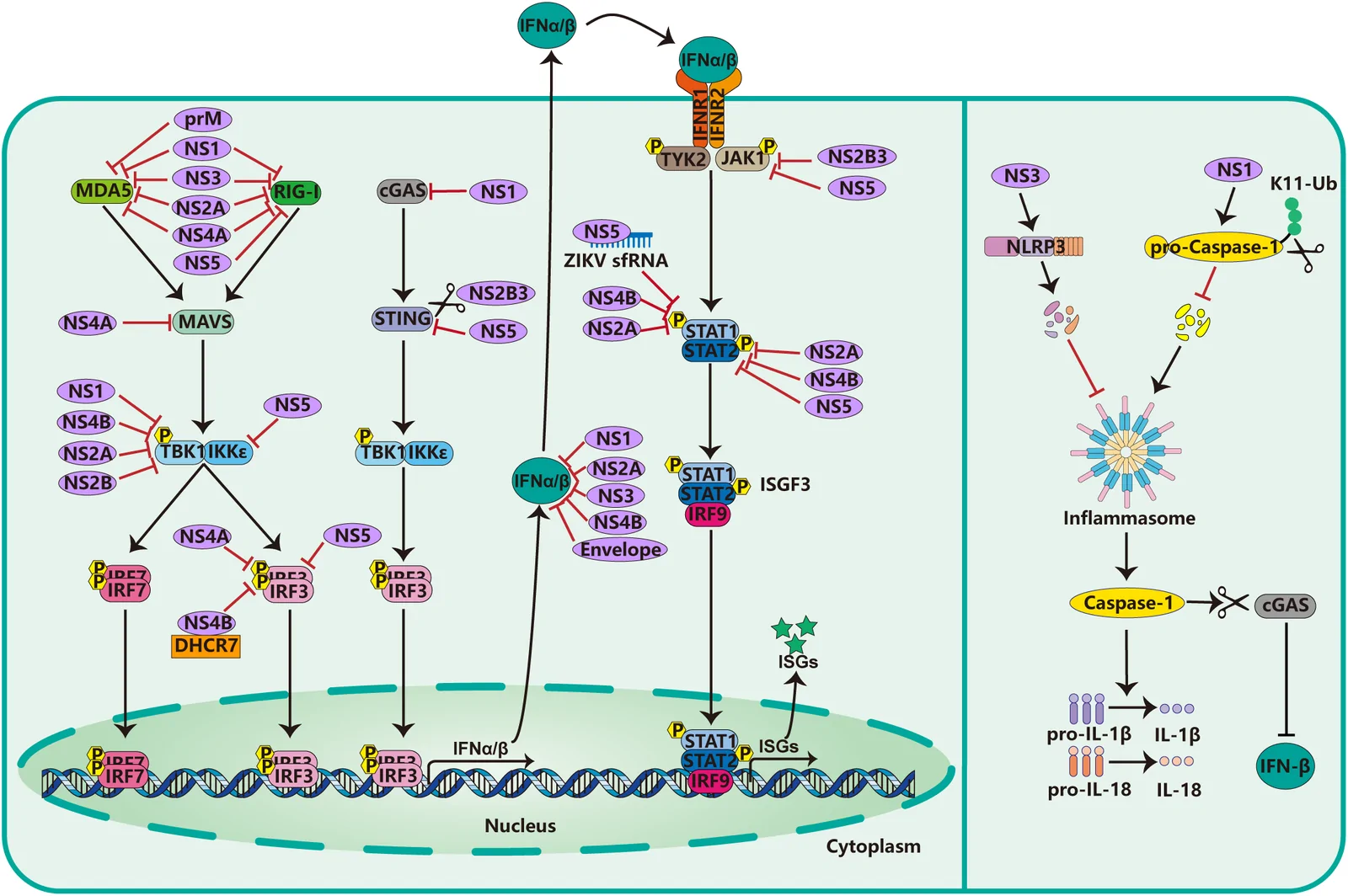
ZIKV evades the immune response by targeting key signaling pathways. In the RLR-MAVS pathway, ZIKV prM inhibits MDA5-mediated signaling, whereas NS1 and NS3 antagonize RIG-I/MDA5 signaling. Downstream, NS2A and NS4A broadly suppress multiple components of the RIG-I/MDA5 pathway; NS1 and NS4B inhibit TBK1 oligomerization; NS2A, NS2B, and NS4B suppress TBK1 phosphorylation; and NS4A and NS5 inhibit IRF3 phosphorylation and activation. NS5 additionally suppresses IKKϵ expression and phosphorylation, thereby blocking IRF3 phosphorylation, while NS4B impairs IRF3 activation through DHCR7. NS4A also induces mitophagy by upregulating mitophagy receptors, leading to reduced MAVS expression. In parallel, NS1 promotes TRAF6 degradation, whereas E, NS2A, NS3, and NS4B cooperatively induce autophagic degradation of AXL/TIM-1, thereby suppressing IFN-β production. In the cGAS-STING pathway, ZIKV disrupts STING signaling through multiple mechanisms, including caspase-1-dependent cleavage, species-specific cleavage by the NS2B3 protease, and NS5-mediated polyubiquitination and degradation of STING, ultimately reducing IFN-I production. In the NLRP3 inflammasome pathway, ZIKV exerts context-dependent effects, with both activation and suppression reported across different viral strains and cellular models; NS3-mediated degradation of NLRP3 represents one mechanism of inflammasome inhibition. Regarding the JAK-STAT signaling pathway, NS2B3 promotes proteasomal degradation of JAK1; NS5-bound HSP90 induces degradation of JAK1 and JAK2; NS2A promotes proteasomal degradation of STAT1 and STAT2; NS4B inhibits STAT1 phosphorylation and STAT2 nuclear translocation; sfRNA stabilizes NS5 to further suppress STAT1 phosphorylation; and NS5 promotes proteasomal degradation of STAT2.

Overall, ZIKV employs multiple coordinated strategies to evade innate immune surveillance, including direct interference with RIG-I/MDA5 and cGAS-STING signaling, disruption of key adaptors and kinases, and modulation of autophagy. Conflicting reports on NLRP3 inflammasome regulation illustrate the importance of context—encompassing cell type, viral strain, and species—in determining infection outcomes. Studies using primary cells *in vitro* (Li et al.; Gim et al.) reported NLRP3 suppression, whereas Wang and He demonstrated NLRP3 activation across multiple primary-cell systems and mouse models, which may better reflect the inflammatory phenotype in an intact host. Viral strain differences may partly account for this discrepancy, as MR766 is a highly passaged African-lineage prototype, whereas z16006 and SZ01 are Asian-lineage isolates associated with recent outbreaks. However, opposing results obtained with the same SZ01 strain (Li vs. He) suggest that strain alone is insufficient, pointing to the contribution of cell-type-specific expression of NLRP3 pathway components and differential priming signals. Additionally, species-specific differences must be considered: ZIKV NS5 degrades human STAT2 but not mouse STAT2, and NS2B3 cleaves human STING but not murine STING, limiting the direct translatability of mouse model findings to human disease. Although inhibitory mechanisms may operate under certain conditions, multi-model and *in vivo* evidence more strongly supports ZIKV-induced NLRP3 activation as the predominant inflammatory outcome. Future research should integrate tissue-specific immune environments (e.g., placenta, brain, testes) and leverage humanized or physiologically relevant models to better dissect the context-dependent interplay between viral evasion strategies and host defenses, and to explore targeting key protein-protein interaction interfaces as a potential antiviral approach.

### 3.2 Antagonism of the JAK-STAT pathway

IFN-I binds to IFNAR1/2 receptors, triggering phosphorylation of JAK1 and tyrosine kinase 2 (TYK2). This recruits and phosphorylates STAT1 and STAT2, which then dimerize, associate with IRF9, and form the interferon-stimulated gene factor 3 (ISGF3) complex. ISGF3 translocates to the nucleus and induces ISG transcription, establishing an antiviral state (143, 144). ZIKV disrupts this signaling cascade through multiple mechanisms.

#### 3.2.1 Targeted disruption of JAK kinases

ZIKV targets JAK1 to block downstream signaling. Wu et al. showed that ZIKV NS2B3 interacts with endogenous JAK1 and promotes its proteasomal degradation, thereby disrupting IFN receptor signaling (121). Roby et al. revealed that heat shock protein 90 (HSP90) is required for maintaining JAK kinase activity. ZIKV NS5 binds to HSP90, inducing the degradation of JAK1 and JAK2, which abrogates downstream IFN responses (145).

#### 3.2.2 Multifaceted interference strategies targeting STAT1 and STAT2 signaling

ZIKV employs multiple strategies to target STAT1 and STAT2, thereby suppressing IFN responses. Fanunza et al. reported that ZIKV NS2A mediates STAT1 and STAT2 degradation via the proteasome pathway (146). ZIKV NS4B indirectly inhibits nuclear translocation of phosphorylated STAT2 by suppressing STAT1 phosphorylation (147). In addition, ZIKV sfRNA binds to NS5, enhancing NS5 stability and accumulation, which intensifies inhibition of STAT1 phosphorylation and blocks antiviral signaling (148).

Similar to DENV and YFV NS5 (149, 150), several studies have reported that ZIKV NS5 binds STAT2 and promotes its proteasomal degradation, thereby suppressing IFN-I signaling (45, 151, 152). Wang et al. further demonstrated that the coiled-coil domain of STAT2 lies at the interface between the MTase and RdRp domains of NS5, and that NS5 binding disrupts STAT2 interaction with IRF9, thereby preventing ISGF3 assembly (153). Peng et al. identified K28 in the MTase domain of NS5 as a key residue involved in both STAT2 degradation and guanylyltransferase activity. This residue also facilitates 5′ capping of viral RNA, linking viral replication to immune evasion (154). Notably, Shu et al. observed that ZIKV infection reduces STAT2 protein levels more efficiently than NS5 overexpression alone, suggesting additional NS5-independent mechanisms. They found that ZIKV infection accelerates the ubiquitin-proteasome degradation of short-lived antiviral proteins, including STAT2, by inhibiting host protein synthesis, thereby synergizing with NS5 to promote immune evasion (155). Recent studies have shown that ZIKV NS5 inhibits IFN signaling via CRL3 E3 ubiquitin ligase-mediated degradation of STAT2 (156) (**Figure 3**).

Overall, ZIKV targets the JAK-STAT pathway through multiple viral proteins that degrade JAK1 and STAT2, inhibit STAT1 phosphorylation, and block STAT2 nuclear translocation. ZIKV also accelerates STAT2 degradation by suppressing host protein synthesis. These synergistic actions disrupt IFN signaling and promote immune evasion. This multi-level strategy, together with suppression of the RLR and cGAS-STING pathways, forms a complex innate immune blockade. Notably, NS5 acts as a central effector, targeting the NS5-STAT2 interface may restore IFN responses. Future studies should examine the dynamic regulation and synergy of these evasion mechanisms across cell types and infection stages.

### 3.3 Subversion of cellular intrinsic mechanisms

#### 3.3.1 ZIKV inhibits NMD

The NMD pathway maintains immune homeostasis by surveilling and degrading aberrant mRNAs. NMD is initiated by up-frameshift 1 (UPF1) phosphorylation and its recruitment downstream of the termination codon. This phosphorylation triggers remodeling of NMD substrates, suppressing translation initiation and recruiting mRNA degradation components (157, 158). Nevertheless, ZIKV has evolved an immune evasion mechanism targeting the NMD pathway. Fontaine et al. demonstrated that ZIKV C protein directly interacts with UPF1, leading to selective proteasomal degradation of nuclear UPF1 and consequent attenuation of NMD-mediated immune defense, which contributes to microcephaly and other neurological disorders (159).

#### 3.3.2 Viral suppressors of RNAi

ZIKV actively suppresses the RNAi pathway by expressing VSRs. Qiu et al. showed that NS2A functions as a VSR, similar to other orthoflaviviruses (160). Zeng et al. found that the C protein directly binds Dicer and inhibits its ribonuclease activity, which blocks the biogenesis of host miRNAs essential for neurodevelopment and leads to impaired neurogenesis and cortical malformation (161). The same group further demonstrated that viral interfering RNA (viRNA) derived from ZIKV mRNA depends on Dicer activity and has antiviral properties in neural stem cells (NSCs), but the C protein as a VSR suppresses Dicer-mediated viRNA production, thereby evading RNAi-mediated immunity and facilitating viral replication (162). In addition to viral proteins, sfRNA also functions as a VSR. Chen et al. first discovered that ZIKV-infected hNPCs produce abundant sfRNA, which competes with siRNA for binding to RISC components, thereby inhibiting RISC formation, suppressing RNAi, and promoting ZIKV replication (163).

#### 3.3.3 ZIKV inhibits SGs

The cellular stress response pathway constitutes a critical defense mechanism against viral infections. Its activation leads to global translational arrest and SG assembly, which sequester translation initiation factors and stalled mRNAs at the initiation phase of translation to modulate stress responses and antiviral activities (164). Key SG nucleators include Ras-Gap-SH3 domain-binding protein 1 (G3BP1), cytoplasmic activation/proliferation-associated protein-1 (Caprin-1), T-cell intracellular antigen-1 (TIA-1), and TIA-1-related protein (TIAR). Studies on other orthoflaviviruses have shown that WNV and DENV NS3 proteins, as well as dsRNA, interact with TIA-1 and TIAR to promote viral RNA synthesis while inhibiting SG formation (165). JEV core protein suppresses SG assembly and enhances viral dissemination through interaction with Caprin-1 (166). Consistent with these orthoflaviviruses, ZIKV has evolved multifaceted strategies targeting SGs—a pivotal host defense—circumventing translational repression and immune constraints. These strategies primarily encompass two facets: direct hijacking of core SG assembly proteins and modulation of host phosphorylation signaling to inhibit SG formation.

Regarding hijacking of SG core proteins, Hou et al. reported that ZIKV infection activates the unfolded protein response, protein kinase R, and eIF2α phosphorylation, leading to translational arrest and triggering stress pathways, but SG formation is suppressed by viral C protein, NS3, and NS4A. ZIKV forms stable complexes through interactions between its RNA and G3BP1 as well as between C protein and G3BP1/Caprin-1, hijacking SG components to prevent complete SG assembly and promote viral replication (167). Bonenfant et al. found that ZIKV infection inhibits SGs formation, with G3BP1 colocalizing with dsRNA and E protein, thereby enhancing ZIKV genome replication and possibly promoting viral particle assembly (168).

ZIKV also interferes with host signaling to block SG formation. Amorim et al. showed that ZIKV infection inhibits ER stress-induced eIF2α phosphorylation, preventing SG assembly and creating a replication-favorable environment (169). Wu et al. demonstrated that NS2B acts as a molecular adaptor, colocalizing with protein phosphatase 1α (PP1α) and eIF2α and enhancing their interaction, which promotes eIF2α dephosphorylation in a PP1α-dependent manner and inhibits SG assembly. NS2B and PP1α also stabilize each other by blocking K48-linked ubiquitination, collectively sustaining eIF2α dephosphorylation and SG suppression to facilitate viral replication. Notably, although PP1α can induce IFN production, NS2B-mediated IFN inhibition occurs independently of PP1α (170).

#### 3.3.4 ZIKV hijacks the host UPR and ER chaperones

Rather than merely acting as a passive inducer of the UPR, ZIKV selectively remodels downstream effector cascades to foster a cellular environment favorable for viral replication. Turpin et al. demonstrated that ZIKV downregulates GRP78/BiP to hijack the UPR and subvert host antiviral defenses (171). Further studies revealed that ZIKV dynamically regulates the host UPR in a time-dependent manner: early PERK and ATF6 co-activation supports viral replication while suppressing GRP78/BiP, whereas late-stage ATF6 engagement and sustained eIF2α phosphorylation drive host translation shutoff and viral release (172). Additionally, ZIKV hijacks host ER chaperones to facilitate viral maturation and replication. As shown by Khongwichit et al., the interaction between ZIKV E protein and GRP78 promotes viral internalization and is essential for robust replication (173), with subsequent findings revealing a concomitant interaction between NS1 and GRP78 (174). Moreover, although CHOP typically mediates ER stress-induced apoptosis, ZIKV can also interfere with CHOP expression to delay apoptotic cell death (175).

#### 3.3.5 ZIKV evasion of apoptosis

ZIKV both induces host cell apoptosis and modulates apoptotic pathways to evade immune detection and promote replication. Wu et al. found that ZIKV NS2B3 inhibits cleavage of caspase-3 and PARP, thereby inhibiting apoptosis and potentially promoting viral replication (121). Additionally, ZIKV inhibits apoptosis through its sfRNA. Slonchak et al. reported that sfRNA prevents caspase-7 upregulation in mosquito host cells, suppressing apoptosis and promoting efficient viral infection and dissemination. Consistent with this, apoptosis inhibition rescued replication of sfRNA-deficient ZIKV mutants (176). Beyond direct viral suppression, host factors also regulate this process. Stokes et al. found that blocking IFN-β significantly increases activated caspase-3, indicating that IFN-β limits ZIKV-induced apoptosis in neural cells (177). Current research explores inhibiting apoptosis pathways to reduce ZIKV-induced neurological damage. Emricasan blocks ZIKV-triggered caspase-3 activity, protecting hNPCs (178), while memantine prevents neuronal death (179), both show neuroprotective effects. Thus, targeting apoptosis pathways is a promising therapeutic strategy for ZIKV-related neurological injury.

#### 3.3.6 Autophagy-mediated ZIKV immune evasion

Autophagy normally acts as a host defense against viral infections, but ZIKV manipulates this pathway to promote replication and evade immunity. The regulation is temporally specific. Sahoo et al. found that in neurons and glial cells, autophagy markers p62 and LC3-II increase early in infection, activating autophagy and reducing viral protein accumulation. However, in late-stage infection, ZIKV inhibits autophagy by activating mTORC1 and mTORC2 and phosphorylating ULK1, promoting viral protein accumulation and replication (180). This time-dependent shift from activation to inhibition is a crucial adaptive strategy that enables ZIKV to evade immune system and replicate efficiently.

Beyond non-selective autophagy, ZIKV also suppresses host defenses mediated by selective autophagy. Zhou et al. reported that ZIKV NS2B3 cleaves p62, reducing p62-mediated degradation of viral NS3 and NS5, thereby promoting ZIKV replication (122). Regarding ER autophagy (ER-phagy), Lennemann et al. showed that the ER-phagy receptor FAM134B limits ZIKV infection, but NS2B3 cleaves FAM134B to inhibit this antiviral function and reticulophagy, thereby facilitating replication vesicle formation and genomic replication (181, 182). Zhang et al. further found that NS2A is ubiquitinated by the E3 ligase AMFR, then binds FAM134B and promotes its proteasomal degradation, disrupting ER-phagy independently of NS2B3 (183, 184). Tiwari et al. found that NS4A evades virophagy and mitophagy by downregulating the selective autophagy gene Fanconi anemia protein (FANCC), contributing to microcephaly by slowing neuronal growth (185).

Collectively, ZIKV systematically subverts host intrinsic defenses by weakening NMD via UPF1 degradation, blocking RNAi via VSRs, inhibiting SG formation, reprogramming the UPR and hijacking ER chaperones, suppressing apoptosis, and temporally regulating autophagy while disrupting selective autophagy receptors. These actions collectively promote viral replication and neuropathogenicity. The seemingly opposing effects of ZIKV on RNAi, UPR, apoptosis, and autophagy reflect context-dependent adaptations governed by infection stage, cell type, and host species. ZIKV antagonism of RNAi represents a host- and cell-type-specific adaptation. Essential for viral persistence against mosquito defenses and effective in mammalian stem cell niches, RNAi suppression becomes secondary in differentiated mammalian somatic cells where interferon signaling predominates. Rather than uniformly activating or suppressing the UPR, ZIKV finely tunes this pathway in a spatiotemporally dynamic and branch-specific manner. This context-dependent regulation strategically balances the co-option of ER stress machinery for viral replication against the mitigation of detrimental consequences, such as severe translational arrest and premature apoptotic clearance. Although context-dependent anti-apoptotic signals exist, compelling evidence indicates that ZIKV predominantly induces apoptosis. Driven by viral components (C, E, NS4B, NS5, sfRNA) via intrinsic, extrinsic, and ER-stress pathways, pro-apoptotic activity directly underlies neuropathology. Crucially, reverse-genetics studies confirm that the prM-S139N mutation heightens NPC apoptosis and microcephaly, establishing apoptosis as a genetically encoded driver of ZIKV pathogenicity. Similarly, while ZIKV transiently hijacks early autophagy for replication, its primary strategy is evading autophagic clearance. Mutant infectious clones demonstrate that disrupting NS2B3- and NS2A-mediated cleavage or ubiquitination abolishes p62 and FAM134B degradation, restoring host autophagic clearance and markedly suppressing viral replication—proving that disabling selective autophagy is a genuine determinant of viral fitness. Future research integrating mutant infectious clones with single-cell spatial transcriptomics, and CRISPR screens in organoids and *in vivo* models will provide deeper spatiotemporal, mechanistic, and structural insights into the interplay among viral genetic variations, host-pathway regulation, and disease severity (**Table 2**).

**Table 2:** Summary of ZIKV subversion of cellular intrinsic mechanisms.

Cellular intrinsic mechanisms	Effector	Target	Mechanism of action	Reference
NMD	C protein	UPF1	Binds and degrades UPF1 via proteasome, weakens NMD defense	(159)
RNAi	NS2A	RNAi pathway	Suppresses RNAi as a VSR	(160)
	C protein	viRNA	Suppresses viRNA production, evades RNAi	(162)
	sfRNA	RISC	Competes with siRNA, blocks RISC formation and RNAi	(163)
SG	C, NS3, NS4A	SG	Block SG assembly	(167)
	RNA	G3BP1	Hijacks SG components, blocks SG assembly	
	C protein	G3BP1, Caprin-1	Hijacks SG components, blocks SG assembly	
	RNA, E protein	G3BP1	Hijack SG components, block SG assembly	(168)
	ZIKV	eIF2α	Inhibits eIF2α phosphorylation, blocks SG assembly	(169)
	NS2B	PP1α, eIF2α	Promotes eIF2α dephosphorylation, inhibits SG assembly	(170)
UPR	ZIKV	GRP78/BiP	Downregulates GRP78/BiP	(171)
	ZIKV	PERK/ATF6	Promotes early viral replication	(172)
		ATF6/eIF2α	Induces translational shutoff and viral release	
	E	GRP78	Facilitates viral internalization	(173)
	NS1	GRP78	Exploits ER chaperone function	(174)
	ZIKV	CHOP	Delays apoptosis	(175)
Apoptosis	NS2B3	caspase-3, PARP	Inhibits cleavage of caspase-3 and PARP, inhibits apoptosis	(121)
	sfRNA	caspase-7	Prevents caspase-7 upregulation, inhibits apoptosis	(176)
	ZIKV induced IFN-β	caspase-3	Prevents caspase-3 activation, inhibits apoptosis.	(177)
Autophagy	ZIKV (early infection)	p62, LC3-II	Activates autophagy, reduces viral protein accumulation	(180)
	ZIKV (late infection)	mTORC1/2	Inhibits autophagy (activates mTORC1/2, phosphorylates ULK1), promotes viral replication	
	NS2B3	p62	Cleaves p62, reduces NS3/NS5 degradation	(122)
	NS2B3	FAM134B	Cleaves FAM134B, inhibits ER-phagy	(182)
	NS2A	FAM134B	Binds and degrades FAM134B, disrupts ER-phagy	(183, 184)
	NS4A	FANCC	Downregulates FANCC, evades virophagy and mitophagy	(185)

## 4 Conclusion and perspectives

ZIKV has emerged as one of the most biologically distinctive and clinically consequential members of the genus *Orthoflavivirus*. Its capacity to cross the placental barrier, persist in the reproductive tract, and cause congenital and neurological disease reflects a level of host adaptation that extends beyond that of most related arboviruses. Central to this success is the virus’s ability to engage the innate immune system in a highly dynamic manner—eliciting robust antiviral responses while simultaneously neutralizing their protective effects.

This review highlights that ZIKV employs a remarkably multifaceted immune evasion strategy. Multiple nonstructural proteins, including NS1, NS2A, NS2B, NS4A, NS4B, and NS5, converge on key signaling pathways—often targeting distinct components within the RIG-I-MAVS, cGAS-STING, and JAK-STAT cascades—while simultaneously subverting cellular processes such as autophagy, apoptosis, and SG formation. This coordinated attack, rather than relying on a single mechanism, provides the virus with both functional redundancy and the flexibility to manipulate host cells in a context-dependent manner.

Importantly, innate immune responses also provide the foundation for subsequent adaptive immunity. Early during infection, IFN-I-mediated innate immunity provides the first line of defense against viral replication and dissemination while enhancing antigen presentation and shaping subsequent T- and B-cell responses. In immunocompetent mice with intact IFN-I signaling, innate immunity plays a dominant role in early viral control; however, when IFNAR signaling is impaired, T-cell and antibody responses become critical for limiting viral dissemination to the brain and testes and for reducing disease severity (186). CD4^+^ T cells contribute to viral control through cytokine production, support of antibody responses, and regulation of CD8^+^ T-cell function (187), whereas B cells generate neutralizing antibodies targeting the ZIKV E protein, including epitopes within EDIII, to block viral entry (188). Thus, innate and adaptive immunity should not be considered independent layers of host defense, but rather interconnected components of antiviral protection. However, adaptive immunity is also context-dependent: prior orthoflavivirus exposure may induce cross-reactive responses that provide heterologous protection, whereas weakly neutralizing antibodies have the potential to enhance ZIKV infection through Fcγ receptor-mediated antibody-dependent enhancement (ADE) (189). Together, these findings highlight the importance of the interplay between rapid innate antiviral responses and the specificity and quality of adaptive immunity in determining the outcome of ZIKV infection.

At the same time, many of the most important questions in the field remain unresolved. It is still unclear why ZIKV, but not most other orthoflaviviruses, exhibits pronounced teratogenicity and long-term persistence in reproductive tissues. The extent to which innate immune responses contribute to protection versus immunopathology in the placenta, developing brain, and testes remains incompletely understood. Moreover, the molecular determinants that govern whether pathways such as autophagy and apoptosis act in an antiviral or proviral capacity are likely highly context-dependent. Addressing these questions will require experimental systems that more faithfully recapitulate human tissue biology, including organoids, placenta-on-a-chip platforms, and humanized animal models.

These mechanistic insights also highlight several therapeutic opportunities. Viral proteins such as NS5 and the NS2B3 protease remain attractive direct-acting targets, although prolonged treatment may select for resistant variants. Inhibitors targeting NS1 and structural proteins, including E, prM, and C, remain limited, underscoring the need for structure-guided target discovery and broad-spectrum anti-flaviviral agents (190). Beyond direct molecular antagonism of innate signaling, evidence from related orthoflaviviruses (such as WNV, TBEV, and DENV) demonstrates that viral replication complexes are physically sequestered within ER-derived invaginated vesicles. This spatial shielding hides viral replication intermediates, particularly dsRNA, from cytosolic PRRs like RIG-I and MDA5 (191–195). Although direct structural and mechanistic validation in ZIKV warrants further investigation, this physical evasion paradigm is likely conserved and suggests that pharmacologically disrupting replication organelle architecture or host-mediated vesicular dynamics could expose the hidden viral genome to intrinsic immunosurveillance. Building upon the viral exploitation of host intracellular membranes, host-directed interventions targeting factors essential for membrane remodeling and vesicular trafficking offer promising avenues to counter viral subversion.

Vaccine development remains a global priority but is challenged by pre-existing orthoflavivirus immunity and the risk of ADE, particularly in individuals with prior orthoflavivirus exposure and in endemic settings (196, 197). Identifying virus-host interfaces that are essential for immune evasion but dispensable for normal cellular function may therefore guide the development of safer host-directed antivirals. Clinical translation requires balancing efficacy, resistance, and host safety, minimizing ADE through rational antigen design, and validating candidate interventions through physiologically relevant models and well-designed clinical studies.

In addition to conventional antiviral approaches, emerging strategies that exploit viral immune-evasion mechanisms may provide alternative therapeutic opportunities. Notably, Li et al. recently established the concept of viral protease-initiated lytic cell death (VID) and designed a novel lipid nanoparticle-encapsulated VID activator mRNA (LNP-VIDA mRNA) platform. This approach exploits the ZIKV NS2B3 protease as a specific switch to induce host cell pyroptosis, combining the targeted elimination of infected cells with immune activation to provide a fast-iterating strategy against viral resistance and emerging orthoflaviviruses (198).

Beyond these therapeutic opportunities, dissecting ZIKV-host interactions reveals general principles of viral immune evasion. As a paradigm for how an emerging RNA virus orchestrates innate immune activation and evasion, ZIKV provides both a mechanistic blueprint for its own pathogenesis and a conceptual framework that can be extended to other orthoflaviviruses and related RNA pathogens. This framework highlights shared principles—including the functional redundancy of viral antagonists, context-dependent pathway outcomes, and the balance between protection and immunopathology—offering broader lessons for combating emerging RNA viruses. Importantly, the interplay between innate and adaptive immunity further emphasizes that the outcome of ZIKV infection is determined not by a single immune pathway, but by the coordinated actions of multiple host defense mechanisms.

Looking ahead, emerging technologies can now be used to test and expand these principles across viral systems. Single-cell and spatial multi-omics, CRISPR-based functional genomics, and comparative analyses across orthoflaviviruses are poised to transform our understanding of ZIKV-host interactions. Integrating these technologies within a coherent conceptual framework will help define the molecular logic that governs immune sensing, the transition from innate to adaptive immunity, tissue tropism, and pathogenesis. Such efforts should not only accelerate the development of effective vaccines and antiviral therapies for ZIKV but also yield broader insights into the principles by which emerging RNA viruses exploit and evade innate immunity.
